# Socioeconomic Inequalities in Health and Perceived Unmet Needs for Healthcare among the Elderly in Germany

**DOI:** 10.3390/ijerph14101127

**Published:** 2017-09-26

**Authors:** Jens Hoebel, Alexander Rommel, Sara Lena Schröder, Judith Fuchs, Enno Nowossadeck, Thomas Lampert

**Affiliations:** 1 Division of Social Determinants of Health, Department of Epidemiology and Health Monitoring, Robert Koch Institute, General-Pape-Straße 62–66, 12101 Berlin, Germany; e.nowossadeck@rki.de (E.N.); t.lampert@rki.de (T.L.); 2 Division of Health Reporting, Department of Epidemiology and Health Monitoring, Robert Koch Institute, General-Pape-Straße 62-66, 12101 Berlin, Germany; a.rommel@rki.de; 3 Institute of Medical Sociology, Martin Luther University Halle-Wittenberg, Magdeburger Straße 8, 06112 Halle, Germany; sara.schroeder@medizin.uni-halle.de; 4 Division of Physical Health, Department of Epidemiology and Health Monitoring, Robert Koch Institute, General-Pape-Straße 62-66, 12101 Berlin, Germany; j.fuchs@rki.de

**Keywords:** socioeconomic position, health inequalities, social determinants, health disparities, unmet needs, access to health services, healthy ageing

## Abstract

Research into health inequalities in the elderly population of Germany is relatively scarce. This study examines socioeconomic inequalities in health and perceived unmet needs for healthcare and explores the dynamics of health inequalities with age among elderly people in Germany. Data were derived from the Robert Koch Institute’s cross-sectional German Health Update study. The sample was restricted to participants aged 50–85 years (*n* = 11,811). Socioeconomic status (SES) was measured based on education, (former) occupation, and income. Odds ratios and prevalence differences were estimated using logistic regression and linear probability models, respectively. Our results show that self-reported health problems were more prevalent among men and women with lower SES. The extent of SES-related health inequalities decreased at older ages, predominantly among men. Although the prevalence of perceived unmet needs for healthcare was low overall, low SES was associated with higher perceptions of unmet needs in both sexes and for several kinds of health services. In conclusion, socioeconomic inequalities in health exist in a late working age and early retirement but may narrow at older ages, particularly among men. Socially disadvantaged elderly people perceive greater barriers to accessing healthcare services than those who are better off.

## 1. Introduction

Over the last decades, public-health research has been paying increasing attention to the social determinants of health [1,2,3]. A wide range of studies consistently show that people from lower socioeconomic groups experience poorer health, have an increased risk of chronic disease, and die at younger ages than those from higher socioeconomic groups [4,5,6,7,8]. For a long time, research on the social gradient in health concentrated primarily on the working-age population, because the workplace was widely regarded as the main source of health inequalities. However, gerontological and epidemiological life-course concepts work on the assumption that exposures or socioeconomic circumstances in early phases of life continue to influence health development, the ageing process, and the risk of chronic diseases and functional impairment in later life [9,10,11,12,13].

Although less attention has been paid to later life compared to other life phases, there has been increasing research activity into health inequalities in older populations in the last years. Studies from high-income countries show that socioeconomic differences in health, morbidity, healthcare, and mortality also exist in older age groups to the detriment of lower socioeconomic groups [14,15,16,17,18,19,20,21,22]. Concerning the extent of health inequalities and their dynamics over the later life course, three contradictory hypotheses are discussed in the literature. While one hypothesis postulates that health inequalities continue largely unchanged in later life (‘continuity’), others assume that health inequalities decline (‘convergence’) or even increase (‘divergence’) at older ages [23,24,25,26]. The hypotheses predominantly refer to the second half of life and focus on the development of health inequalities across the transition from middle age to old age. Up to now, empirical research has provided no consistent evidence with regard to the veracity of these hypotheses [18,19,24,27,28,29,30,31,32,33,34].

The present study focuses on socioeconomic differences in health and subjectively perceived financial access barriers to healthcare among Germany’s elderly population. The statutory retirement age in Germany is currently being increased gradually from 65 to 67 years; however, in the older cohorts, it is still predominantly 65 years. Approximately 21 percent of the population of Germany are currently 65 years of age or older [35]. The aims of the present study were to examine: (1) whether socioeconomic inequalities in health and perceived unmet needs for healthcare exist in the elderly population of Germany; and (2) whether the extent of socioeconomic inequalities in health remains constant (continuity), narrows (convergence), or widens (divergence) with age among the elderly in Germany. By exploring these questions, our study aims to give an overview of health inequalities in the older population of Germany. The findings can contribute to the growing body of research on socioeconomic inequalities in health and healthcare in older populations, as well as to understanding the dynamic of health inequalities in the second half of life.

## 2. Materials and Methods

### 2.1. Study Design and Data Collection

The analyses presented in this article were based on data derived from the cross-sectional German Health Update (GEDA) study, a national health survey of the adult population in Germany. GEDA is part of the German health monitoring system administered by the Robert Koch Institute (RKI) in Berlin [36]. The RKI is a federal institution within the portfolio of the German Federal Ministry of Health. The aim of the regularly conducted GEDA surveys is to provide current data on population health, health determinants, and the use of health services [37,38].

For the present study, we used data from the 2014/15 wave of the GEDA study, which was hosting the data collection for wave 2 of the European Health Interview Survey (EHIS) in Germany (GEDA 2014/2015-EHIS). The survey was based on a two-stage stratified cluster sample. The target population was men and women with permanent residence in Germany. In the first sampling stage, 301 communities were randomly selected as primary sampling units from a list of all the municipalities in Germany, stratified by administrative districts and the ‘BIK region size classes’ [39], which take account of the population size, as well as the regional population and employment density. Sampling probabilities were proportional to the population size of the municipalities using the Cox procedure for controlled rounding [40]. In the second sampling stage, people with permanent residence in the sampled municipalities were randomly drawn as secondary sampling units from the local population registers.

Data were collected either by a self-administered postal or web questionnaire. The standardized questionnaires were administered in the German language and included questions about health status, health determinants, use of health services, and sociodemographic characteristics. A total of 24,016 individuals aged 18 years and older had completed the survey between November 2014 and July 2015. For the analysis presented in the current paper, the study population was restricted to elderly participants aged 50–85 years (*n* = 11,811) and divided into two age groups: 50–64 (*n* = 6418) and 65–85 (*n* = 5393). According to the internationally used standard definitions of outcome rates for surveys [41], the ‘Response Rate 1’ was 29.9% in the population aged 50–85 (50–64: 31.3%; 65–85: 28.3%). This response rate is also known as the ‘minimum response rate’ and represents the number of complete interviews divided by the number of interviews plus the number of non-interviews and all cases of unknown eligibility. The study was approved by the Federal Commissioner for Data Protection and Freedom of Information in Germany. Informed consent was obtained from all participants. Participants were informed about the goals and contents of the study, about privacy and data protection, and that their participation in the study was voluntary. Further information on the GEDA 2014/2015-EHIS survey can be found in the study protocol [38].

### 2.2. Socioeconomic Status

Socioeconomic status (SES) was determined using a composite index developed for the national health monitoring system in Germany [42,43]. The index includes information on education, (former) occupation, and income. Education was assessed using the CASMIN (Comparative Analysis of Social Mobility in Industrial Nations) educational classification, which takes into account information on respondents’ school-leaving and vocational qualifications [44]. Occupational status was determined using the International Socio-Economic Index of Occupational Status (ISEI) developed by Ganzeboom et al. [45]. In cases where no person in the household was currently employed (e.g., retirees), the occupation pursued most recently was used. Income level was assessed via the net equivalent income; for this, household net income was adjusted for household size and the age-specific needs of the household members using the modified equivalence scale of the Organisation for Economic Co-operation and Development (OECD) [46]. To calculate the SES index, the three individual dimensions were transferred to metric subscales with a value range of 1.0 to 7.0. The point scores of the three subscales were then summed to compute a total score ranging from 3.0 to 21.0. The total score was classified by quintiles of the total adult population as “low SES” (quintile 1), “middle SES” (quintiles 2–4), or “high SES” (quintile 5). More details on the index and methods used in its construction can be found elsewhere [42,43].

### 2.3. Health Problems

Health problems were assessed by two global and two more specific indicators: less-than-good self-rated health, global activity limitations, walking limitations, and low back complaints. Self-rated health was measured with the question “How is your health in general? Is it (1) very good, (2) good, (3) fair, (4) bad, (5) very bad?” [47,48]. For the analysis, we created a binary variable indicating less-than-good self-rated health (categories 3–5). Dichotomization was necessary as the response options were not equidistant and Brant tests indicated that the parallel regression assumption was violated. Global activity limitations were determined by asking participants whether they are permanently (≥6 months) limited because of a health problem in activities people usually do. The questions on walking limitations were “Do you have difficulty walking half a km (500 m) on level ground that would be the length of five football fields without the use of any aid?” and “Do you have difficulty walking up or down 12 steps?” Those participants who reported any difficulty were considered as having global activity limitations or being limited in walking. Low back complaints were operationalized with the category “Low back disorder or other chronic back defect” after the question “During the past 12 months, have you had any of the following diseases or conditions?”

### 2.4. Unmet Healthcare Needs

Perceived unmet needs for healthcare due to financial access barriers were assessed with the European Healthcare Module [47]. The question asked was “Was there any time in the past 12 months when you needed the following kinds of healthcare, but could not afford it?”, followed by a list of kinds of care: medical care, dental care, prescribed medicines, and mental healthcare (response categories: “yes”, “no”, or “no need”). For each kind of care, we created a binary variable indicating the perceived unmet need. Participants who reported that they had “no need” for the respective kind of care were treated as missing data to exclude them from the analysis and restrict the analyses to the population with a self-assessed need for healthcare. The sample sizes of 50- to 85-year-old people with self-assessed need were *n* = 10,015 for medical care, *n* = 10,162 for dental care, *n* = 9884 for prescribed medicines, and *n* = 6657 for mental healthcare. The frequencies can be found in Table 1, along with the characteristics of the study population.

### 2.5. Covariates

To prevent potential confounding by sociodemographic factors, we included age, residential region, urbanization, and immigrant status as covariates in our analysis. Residential region was taken into account by a variable differentiating between participants living in the western part (old federal states) and those living in the eastern part (new federal states including Berlin) of Germany. Using the district typology of the German Federal Institute for Research on Building, Urban Affairs and Spatial Development, we assigned the administrative districts in which the participants lived to urban and rural areas. Immigrant status was operationalized with a binary variable distinguishing between participants who were born within the current borders of Germany and those who were not. In the analysis of inequalities in unmet needs for healthcare, the type of health insurance (statutory, private, other) was considered as an additional covariate in view of the differences between statutory and private health insurance with regard to access to healthcare and the reimbursement of medical services [49].

### 2.6. Statistical Methods

Inequalities in health and their dynamics with age were analysed in four steps. In step 1, we stratified by age group and computed adjusted odds ratios (OR) according to SES using binomial regression with a logistic link function (logistic regression model). The resulting ORs were used to quantify the extent of relative health inequalities in each age group. In step 2, we fitted a common model including individuals from both age groups and added an interaction term of SES × age group to the model in order to test whether the ORs by SES differed between the age groups. In step 3, we estimated model-adjusted prevalence rates using predictive margins [50] based on the binomial regression analyses in step 2. The model-adjusted prevalence rates were computed according to SES and age in order to model the dynamic of SES-related differences in health with age, while adjusting for covariates. In step 4, we estimated model-adjusted prevalence differences between the SES groups using binomial regression with an identity link function (linear probability model). Prevalence differences represent the extent of absolute health inequalities. We again added an interaction term of SES × age group to the model to test whether the prevalence differences between SES groups varied between age groups. By considering prevalence differences in addition to ORs, our analysis took account of not only relative, but also absolute inequalities in health [51,52]. Considering both kinds of inequalities is particularly indicated when the overall prevalence of a health outcome differs greatly between the groups to be compared. This was the case for some of the health problems considered in our analysis, as their prevalence was higher in the older age group than in the younger age group.

In all the analyses, weighting factors were used to account for unequal sampling probabilities and to adjust the distribution of the net sample by sex, age, education, residential region, federal state, and urbanization grade to match the official German population statistics. The analysis was conducted in STATA 14.1 (StataCorp LP, College Station, TX, USA) using the survey data commands to account for the complex sample design. Each analysis step was performed separately for men and women to identify sex-specific dynamics of health inequalities with age and to prevent potential gender bias. Associations and interactions were considered statistically significant when *p* < 0.05.

## 3. Results

Table 2 shows the unadjusted prevalence rates and adjusted ORs of health problems according to SES, stratified by age group. Less-than-good self-rated health, global activity limitations, walking limitations, and low back complaints were each more prevalent among lower SES groups. This was observed in both men and women aged 50–64, as well as those aged 65–85. After adjusting for covariates, low SES remained significantly associated with higher odds of each of the four health problems in both sexes and age groups. The interaction analysis between SES and age group showed that in men, the ORs of less-than-good self-rated health, global activity limitations, and walking limitations were significantly smaller among older men aged 65–85 than among those aged 50–64 (*p*-values for interaction terms < 0.05). In women, this was only found for global activity limitations and walking limitations. The ORs for low back complaints did not differ significantly between the age groups, either in men or women.

**Figure 1:**
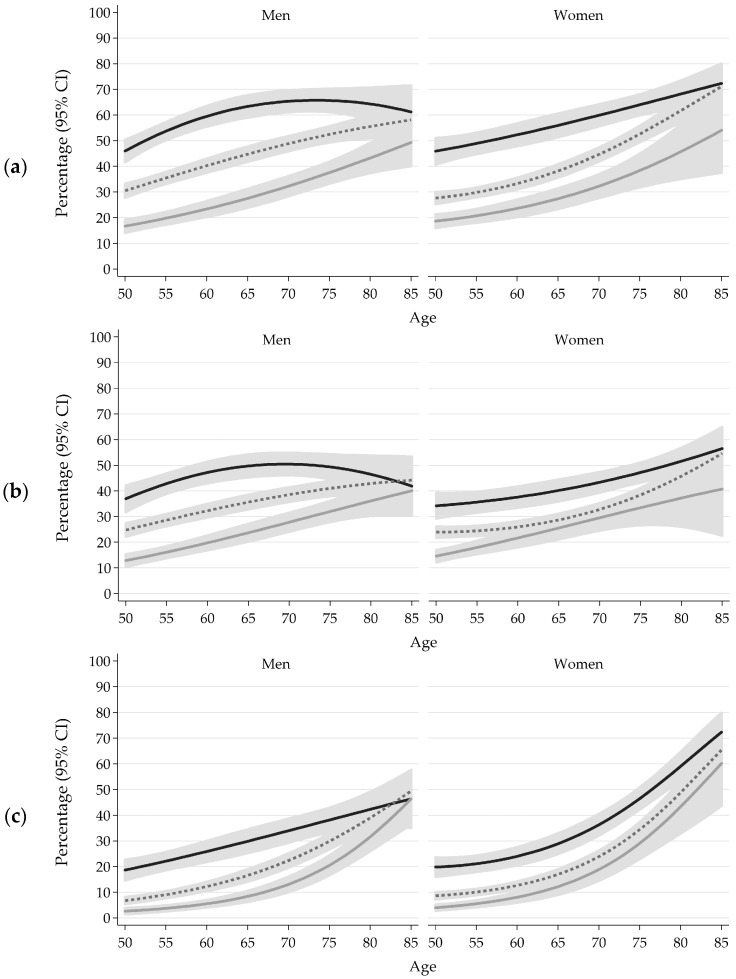

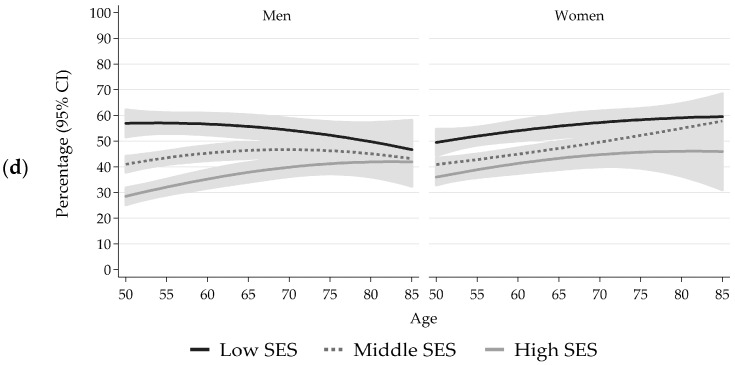
Model-adjusted prevalence with 95% confidence intervals (CI) of less-than-good self-rated health (**a**), global activity limitations (**b**), walking limitations (**c**), and low back complaints (**d**), by socioeconomic status (SES) and age, adjusted for residential region, urban/rural, and immigrant status.

Figure 1 presents the model-adjusted prevalence rates for the four health problems, adjusted for the covariates. Among men, the model-adjusted prevalence differences between the SES groups were significantly reduced in older age groups for less-than-good self-rated health (*p*-value for interaction term = 0.001), global activity limitations (*p* < 0.001), and walking limitations (*p* = 0.002), but not for low back complaints (*p* = 0.222). Among women, the prevalence differences were found to neither decline nor increase with age for less-than-good self-rated health (*p*-values for interaction term = 0.610), global activity limitations (*p* < 0.887), walking limitations (*p* = 0.998), and low back complaints (*p* = 0.075).

Table 3 shows the unadjusted prevalence rates and adjusted ORs of perceived unmet needs for medical care, dental care, prescribed medicines, and mental healthcare according to SES and stratified by age group. The analyses were restricted to people with a self-assessed need for the corresponding healthcare services. Although the prevalence of perceived unmet needs was rather low overall, it was higher among men and women with low SES than among those with middle or high SES. This pattern was observed for medical care, dental care, and prescribed medicines in both age groups. For mental healthcare, the pattern was only apparent in the age group up to 64. After adjusting for age, residential region, urbanization, immigrant status, and type of health insurance, low SES was associated with higher odds of perceived unmet needs for medical care, dental care, prescribed medicines, and mental healthcare in men and women up to the age of 64. In the older group aged 65–85, this association was also found among men for medical care, dental care, and prescribed medicines, as well as among women for prescribed medicines. Significant interactions between SES and age group were, however, only found for unmet needs for dental care among men. For mental healthcare among men, the interaction was marginally non-significant.

## 4. Discussion

The aims of this study were to explore the existence of socioeconomic inequalities in health and perceived unmet needs for healthcare in the elderly population of Germany and to investigate the dynamics of SES-related health inequalities with age in this central European population. Our findings demonstrate that socioeconomic inequalities in health exist not only in the older working-age population, but also in the retired population of Germany. The prevalence of perceptions of unmet needs for healthcare was low overall. However, elderly members of socioeconomically disadvantaged groups perceived greater barriers to healthcare than those who were better off, although these inequalities were more evident in a late working age than after retirement. The results on the dynamics of SES-related health inequalities with age provide evidence for both the continuity and the convergence of inequalities in old age, depending on gender, health indicators, and whether absolute or relative inequalities are considered. We found more evidence of converging health inequalities with age for men than for women, suggesting that convergence may occur at earlier ages among men compared to women.

### 4.1. Strengths and Limitations

One of the strengths of this study is the use of up-to-date data from a relatively large national sample, which allowed separate analyses for men and women. Owing to the sample design and the weighting factors used to adjust for survey non-response, it was possible to draw conclusions for the population of Germany from our results. However, institutionalized persons were under-represented in the sample, which must be acknowledged as a limitation to the representativeness of our data. This particularly applies to the oldest age group in which the institutionalization rate (e.g., people in nursing homes) is higher than in the younger group. Moreover, health problems of older people can play a role in non-participation in health surveys [53], which can lead to an underestimated prevalence of poor health and functional limitations. These aspects should be borne in mind when interpreting the results of our study.

As data were collected using self-administered questionnaires, social desirability bias should have been low, as this type of bias occurs mainly when interviewers are involved in the data-collection process [54,55]. Recall bias should have been low for self-rated health and walking limitations, since the corresponding questions referred to the present. For global activity limitations, low back complaints and unmet needs for healthcare, recall periods were longer at up to twelve months. Especially in the older age group, participants may have had difficulties remembering the exact time period in which possible events occurred, which could have biased our results.

Several other limitations should also be considered when interpreting the results. Due to the observational and cross-sectional nature of our data, we could not infer causality or establish the causal direction of the associations between SES on the one hand, and health problems and unmet needs for healthcare on the other. Further limitations arise from the relatively low response rate. Systematic non-response may have led to selection bias, which could have affected our results. However, as the literature suggests that there is no clear relation between the response rate and representativeness of response [56,57], a low response does not necessarily lead to strong selection or weak external validity in general. With regard to selectivity, it should be further noted that immigrants without German language skills may have been underrepresented in the sample, as the survey was only administered in German. Concerning the statistical analysis, it is worth noting that multiple testing was conducted. As a result, some of the significant results may have occurred by chance.

With regard to the dynamics of health inequalities with age, it should be borne in mind that our cross-sectional analysis was not able to disentangle the effects of age and cohort. Our results therefore only reflect the status quo of the current age groups in the German population and the extent of health inequalities within these groups, but not the longitudinal development of health inequalities within individuals of an ageing cohort. The analysis was stratified by sex to identify sex-specific dynamics of health inequalities with age. In this respect, it must be acknowledged that stratification by sex involves the risk of creating artificial differences between men and women or perceptions of gender differences where there are none. Although our results suggest some differences between men and women in the dynamics of health inequalities with age, these differences should be interpreted with caution.

Particularly when it comes to measuring unmet needs for healthcare caused by financial barriers, it must be borne in mind that the data are based on self-reported information, provided by the study participants, that reflect subjective perceptions of the respondents. As a result, the indicators used provide evidence on subjectively perceived access barriers to the healthcare system, but not on actual access restrictions in an objective case of need, let alone on any withholding of health services in an emergency. However, the participants’ self-reported data do reflect how (potential) users of the healthcare system in Germany assess the possibilities for accessing services from their perspective. In this respect, it should be borne in mind that our results only reflect the unmet needs caused by perceived financial barriers. Unmet needs for healthcare may, however, also arise from other issues, such as language barriers or a lack of information, which are not directly reflected by the indicators of unmet need used in the present study. However, since in Germany the exemption from copayments for prescribed medicines has to be formally applied for, perceived unmet needs due to costs may also indirectly reflect language barriers or a lack of information.

With regard to potential interactions between SES and age group in the prediction of unmet needs for healthcare, it has to be considered that the numbers of respondents included in the analyses (those with self-assessed need for healthcare) differed substantially between the four kinds of services. The lowest number of respondents was available for mental health services (see Section 2.4). Accordingly, failing to detect an interaction that is actually present (type II error) was more likely in the analyses of unmet needs for mental health care than for the other kinds of services. In particular, this can be a reason why the interaction between SES and age group in the prediction of unmet needs for mental health services was not statistically significant, although the SES coefficients for the two age groups were very different.

In the present study, SES was measured on the basis of extensive information on the education, occupation, and income of the study participants. The index used is particularly suitable when analyses aim to provide a broad overview of health inequalities in a population with a view to different health indicators [43,58], as was the aim of the present study. The index takes into account all three core dimensions of vertical social inequality in a single measurement and makes it possible to recognize additive effects of the individual dimensions on health. However, it must be admitted that the index has the disadvantage that the relative importance of the individual dimensions is hidden by the index [59]. Furthermore, it does not take into account the effects of status inconsistencies, i.e., very different positions in the individual dimensions [60]. In future analyses, therefore, the individual indicators of SES could be used to complement our overview by giving us more profound insights into the mechanisms on which the health inequalities are based [61,62,63].

### 4.2. Comparison with Previous Findings and Possible Explanations

The findings of the present study are consistent with a growing body of studies from high-income countries showing that socioeconomic inequalities in health exist not only in middle age, but also in later phases of adulthood [16,17,18,19,20,21,64,65,66,67,68,69,70,71]. With regard to the dynamics of health inequalities with age, our results support previous findings from cross-sectional and longitudinal studies indicating that health inequalities continue to exist at older ages [18,19,27,31], but may be partially smaller than at middle age [18,25,32,33,34]. However, there are also findings which indicate that health inequalities may widen in older age [19,24,27,32]. The literature suggests that results on the dynamics of health inequalities in the course of adulthood may depend, among other factors, on the dimension of health considered [19,32]. This is, in part, also supported by our findings indicating that inequalities in some health problems may decline with age, while inequalities in other health problems may not, or at least not before the age of 85. In this respect, our results suggest that different findings on the convergence and continuity of health inequalities in later life may be related to the age dependency of the health dimension considered. We found evidence for the convergence hypothesis with regard to poor self-rated health, global activity limitations, and walking limitations, each showing a relatively strong age dependency. In contrast, our results suggest the continuity of inequalities in low back complaints, which showed a comparatively low age dependency.

One explanation often given for the continuity of health inequalities in later life is that the SES acquired in earlier life phases does not usually change significantly during the transition from middle to old age (‘status maintenance’) [72], e.g., as a result of the pension systems in modern welfare states. Accordingly, SES can be expected to continuously shape health and life chances in old age to the extent that the health gap between different socioeconomic groups remains constant throughout later life [19,27]. Furthermore, socioeconomic conditions and psychosocial factors during middle age can still have an impact on health chances in old age and thus contribute to health inequalities in old age [69,73].

‘Selective survival’ processes are frequently put forward as explanations of the convergence of socioeconomic health inequalities in old age [74,75]. Selective survival means that lower socioeconomic groups have a higher risk of premature mortality, with the result that a smaller percentage survives to old age. Those people from lower socioeconomic groups who survive to old age are therefore likely to represent a positively selected group in relation to health characteristics, and to differ less in terms of health from higher socioeconomic groups than in middle age. Data from different European countries suggest that mortality declines in all socioeconomic groups, even though the decline may be faster in higher socioeconomic groups [76]. If this trend continues, more and more people—also from lower socioeconomic groups—could reach old age in the future. Part of the decline in premature mortality is due to the fact that an increasing number of people survive potentially life-threatening diseases such as heart attacks, strokes, and diabetes mellitus. However, the survivors are then often affected by secondary diseases and functional limitations [77,78,79,80]. As a result, health inequalities could increasingly last from middle to old age in the future. Then, the convergence of health inequalities among older men could also shift from old age to very old age in the future. However, the extent to which this scenario actually occurs will also depend on the extent to which selective survival really does contribute to the explanation of converging health inequalities in old age; several studies indicate that the contribution is not very large and that selective survival can explain only some of the decline in health inequalities with age [25,28,34,81].

Apart from selective survival, the convergence of health inequalities in old age is also explained with the ‘age-as-a-leveler’ hypothesis, which suggests that the age dependency of health is more pronounced at older ages than at younger ages [26,82]. As a result, health in old age is primarily seen as a result of biological or endogenous ageing processes. For example, longevity depends on certain genetic dispositions [83], which are distributed regardless of socioeconomic characteristics. Following the hypothesis, endogenous ageing processes could increasingly mask the effects of exogenous socioeconomic conditions in old age and thus contribute to a convergence of socioeconomic group differences in health.

As regards socioeconomic differences in a perceived unmet need for healthcare, earlier studies have shown that the prevalence of unmet needs varies greatly by European comparison. The level in Germany corresponds to that of other central and northern European countries and is comparatively low compared to the rest of Europe [84,85,86]. The available studies show a distinct relationship between SES and perceived unmet need, which is consistent with our results. This relationship has also been found in older populations in previous studies [22,87,88]. International data reveal that the association of SES and unmet need is stronger in countries with higher income inequality and lower accessibility of the primary care system [86].

Several studies suggest that low income may be the strongest social predictor of perceived unmet needs [89,90,91,92]. People with low SES may have less money at their disposal to spend on healthcare, and might therefore feel forced to forgo healthcare when allocating their money, although they perceive a need. This could apply especially to healthcare services for which out-of-pocket payments or co-payments have to be made by those who use them, even though they have health insurance [93]. In Germany, this probably applies in particular to dental treatment, especially to dental prostheses, which in Germany are subject to the highest co-payments. By contrast, primary healthcare by doctors and psychologists is free of co-payments. Although there is a co-payment obligation for prescribed medicines in Germany, this is strictly capped, especially for the chronically ill. Nevertheless, one can gain the impression of self-restraint in people’s use of health services. For example, many doctors provide services that are not recognized by the statutory health insurance funds as evidence-based (so-called non-covered individual health services); these must be self-financed. Similarly, there are sometimes quite long waiting times in fields such as psychotherapeutic healthcare [94], which could contribute to the perception of access barriers. In addition, low-SES people may have less knowledge about the structures and services of the healthcare system, as they have lower levels of health literacy, and may also be less confident with symptom interpretation and in communicating with physicians [95,96,97]. As a consequence, they might be more willing to avoid healthcare utilization and to perceive their individual needs to be unmet by physicians and the healthcare system.

## 5. Conclusions

This study contributes to a growing body of research on socioeconomic inequalities in health and healthcare in the elderly populations of developed countries and expands the research on this topic with current findings from Germany. According to the results, socioeconomic inequalities in health exist not only in late working age, but also in later life. Although health inequalities were found to still be existent after retirement, our findings suggest that they narrow at older ages, particularly among men. Further, the findings provide evidence suggesting that elderly people of lower socioeconomic groups perceive greater barriers to access healthcare than the better off. All in all, our findings confirm that population-based measures to promote, maintain, and restore health must take into account the special needs of socially disadvantaged people—not only in younger age groups, but also in the elderly population. As a result of the accumulation of health problems and perceived barriers in access to healthcare, older people from socially disadvantaged groups have a particular need for healthcare and support. This need poses special challenges not only for the healthcare system, but also for informal healthcare outside the professional health system, as well as for the social security systems and the welfare state.

## Figures and Tables

**Table 1:** Characteristics of the study population.

	Men	Women	Total
	(*n* = 5702)	(*n* = 6109)	(*n* = 11,811)
Age, years			
Mean ± SD	63.8 ± 9.7	64.8 ± 9.4	64.3 ± 9.6
Age group, % (*n*)			
50–64 years	55.8 (2952)	51.5 (3466)	53.6 (6418)
65–85 years	44.2 (2750)	48.5 (2643)	46.4 (5393)
Socioeconomic status, % (*n*)			
Low	20.9 (1083)	23.7 (1198)	22.4 (2281)
Medium	55.8 (2884)	63.2 (3626)	59.7 (6510)
High	23.3 (1724)	13.1 (1272)	18.0 (2996)
Health problems, % (*n*)			
SRH (less than good)	43.0 (2344)	43.4 (2443)	43.2 (4787)
Global activity limitations	34.1 (1857)	33.0 (1864)	33.5 (3721)
Walking limitations	18.6 (1019)	24.2 (1251)	21.6 (2270)
Low back complaints	45.4 (2372)	48.8 (2771)	47.2 (5143)
Perceived unmet need, % ^1^ (*n*)			
Medical care	3.1 (142)	4.0 (208)	3.6 (350)
Dental care	8.2 (381)	8.0 (427)	8.1 (808)
Prescribed medicines	2.7 (125)	3.2 (159)	3.0 (284)
Mental healthcare	1.7 (55)	2.4 (83)	2.1 (138)
Residential region, % (*n*)			
West	78.7 (4183)	78.3 (4534)	78.5 (8717)
East	21.3 (1519)	21.7 (1575)	21.5 (3094)
Urbanization, % (*n*)			
Rural area	33.6 (1921)	33.5 (2044)	33.6 (3965)
Urban area	66.4 (3781)	66.5 (4065)	66.4 (7846)
Immigrant status, % (*n*)			
Not immigrated	93.6 (5304)	93.7 (5661)	93.6 (10,965)
Immigrated	6.4 (352)	6.3 (396)	6.4 (748)
Type of health insurance, % (*n*)			
Statutory	72.6 (3931)	82.8 (4799)	77.9 (8730)
Private	25.9 (1552)	16.5 (1120)	21.0 (2672)
Other	1.5 (77)	0.8 (44)	1.1 (121)

% = weighted percentage (extrapolated to the population of Germany); *n* = unweighted number in the sample; ^1^ weighted percentage among those who reported having had a need for the corresponding kind of healthcare (see Section 2.4 for valid case numbers); Mean = weighted mean (extrapolated to the population of Germany); SD = standard deviation; SRH = self-rated health.

**Table 2:** Self-reported health problems among men and women in Germany, by socioeconomic status and age group.

	Self-Rated Health (Less Than Good)		Global Activity Limitations		Walking Limitations		Low Back Complaints	
	%	OR ^1^ (95% CI)	%	OR ^1^ (95% CI)	%	OR ^1^ (95% CI)	%	OR ^1^ (95% CI)
Men (50–64 years)	38.0		30.6		11.8		44.2	
Low SES	58.4	5.12 (3.92–6.70) ***	48.1	4.85 (3.69–6.38) ***	25.4	7.64 (4.94–11.81) ***	58.1	2.61 (1.98–3.46) ***
Middle SES	37.8	2.23 (1.79–2.79) ***	30.7	2.26 (1.78–2.87) ***	10.1	2.50 (1.27–3.84) ***	43.8	1.50 (1.22–1.84) ***
High SES (ref.)	21.6	1.00	16.5	1.00	4.5	1.00	34.0	1.00
Men (65–85 years)	49.4		38.4		27.3		47.0	
Low SES	62.3	3.05 (2.33–4.00) ***	44.9	1.76 (1.34–2.32) ***	34.2	2.27 (1.66–3.11) ***	53.4	1.70 (1.30–2.23) ***
Middle SES	49.9	1.86 (1.46–2.36) ***	38.7	1.39 (1.11–1.74) **	27.8	1.67 (1.27–2.21) ***	47.3	1.36 (1.09–1.68) **
High SES (ref.)	34.7	1.00	31.5	1.00	19.2	1.00	39.9	1.00
*p*-value for SES × age group		(0.028)		(<0.001)		(<0.001)		(0.076)
Women (50–64 years)	35.1		28.2		14.4		45.7	
Low SES	52.3	3.71 (2.79–4.92) ***	39.2	2.54 (1.93–3.35) ***	25.9	4.31 (2.92–6.37) ***	55.2	2.00 (1.56–2.55) ***
Middle SES	33.1	1.74 (1.39–2.18) ***	27.1	1.50 (1.20–1.87) ***	12.8	1.91 (1.34–2.72) ***	44.8	1.31 (1.08–1.57) **
High SES (ref.)	22.3	1.00	19.7	1.00	7.1	1.00	37.8	1.00
Women (65–85 years)	52.4		38.2		34.7		52.3	
Low SES	63.7	2.91 (2.05–4.12) ***	46.2	1.81 (1.24–2.64) **	45.6	2.21 (1.55–3.15) ***	56.5	1.42 (1.02–1.98) *
Middle SES	49.5	1.73 (1.27–2.36) ***	35.8	1.23 (0.89–1.71)	31.3	1.34 (0.96–1.86)	51.2	1.13 (0.85–1.51)
High SES (ref.)	34.6	1.00	29.8	1.00	23.5	1.00	46.9	1.00
*p*-value for SES × age group		(0.104)		(0.015)		(<0.001)		(0.231)

*** *p* < 0.001; ** *p* < 0.01; * *p* < 0.05; SES = socioeconomic status; % = unadjusted prevalence rate; OR = odds ratio; CI = confidence interval; ref. = reference category. ^1^ Adjusted for age, residential region, urban/rural and immigrant status. Note: The ORs are derived from separate regression models (analysis step 1); the *p*-values for the interaction term are derived from a common regression model including both age groups (analysis step 2).

**Table 3:** Perceived unmet healthcare needs among elderly men and women in Germany with self-assessed need for healthcare, by socioeconomic status and age group.

		Unmet Need: Medical Care		Unmet Need: Dental Care		Unmet Need: Prescribed Medicines		Unmet Need: Mental Healthcare
	%	OR ^1^ (95% CI)	%	OR ^1^ (95% CI)	%	OR ^1^ (95% CI)	%	OR ^1^ (95% CI)
Men (50–64 years)	3.2		9.6		2.6		2.4	
Low SES	6.5	2.65 (1.13–6.24) *	12.5	1.91 (1.13–3.22) *	5.0	6.40 (2.09–19.62) **	5.1	11.75 (2.35–58.72) **
Middle SES	2.2	0.84 (0.40–1.75)	10.3	1.72 (1.09–2.73) *	2.5	3.24 (1.21–8.68) *	1.7	3.92 (0.82–18.65)
High SES (ref.)	2.5	1.00	5.7	1.00	0.8	1.00	0.6	1.00
Men (65–85 years)	2.9		6.5		2.9		1.0	
Low SES	4.6	3.83 (1.11–13.25) *	8.5	4.50 (2.19–9.25) ***	5.3	7.83 (2.13–28.75) **	0.8	0.87 (0.13–5.73)
Middle SES	3.0	2.54 (0.78–8.29)	7.4	4.18 (2.18–8.01) ***	2.5	2.75 (0.95–7.99)	1.1	1.23 (0.31–4.94)
High SES (ref.)	0.8	1.00	1.6	1.00	0.9	1.00	0.9	1.00
*p*-value for SES × age group		(0.131)		(0.003)		(0.760)		(0.058)
Women (50–64 years)	5.4		10.6		3.8		3.1	
Low SES	10.2	4.25 (2.28–7.94) ***	16.9	2.48 (1.60–3.84) ***	7.5	3.79 (1.67–8.59) **	6.1	3.43 (1.45–8.13) **
Middle SES	4.6	1.85 (1.04–3.29) *	9.7	1.34 (0.89–2.00)	3.2	1.69 (0.79–3.59)	2.2	1.11 (0.49–2.50)
High SES (ref.)	2.7	1.00	6.4	1.00	1.5	1.00	2.1	1.00
Women (65–85 years)	2.6		5.0		2.6		1.5	
Low SES	4.1	2.07 (0.59–7.21)	7.5	2.15 (0.68–6.77)	4.6	4.45 (1.05–18.85) *	1.5	0.72 (0.12–4.53)
Middle SES	1.8	0.98 (0.29–3.35)	4.0	1.15 (0.38–3.51)	1.9	2.14 (0.50–9.08)	1.6	1.08 (0.19–6.21)
High SES (ref.)	2.3	1.00	3.3	1.00	0.8	1.00	0.9	1.00
*p*-value for SES × age group		(0.258)		(0.696)		(0.507)		(0.392)

*** *p* < 0.001; ** *p* < 0.01; * *p* < 0.05; SES = socioeconomic status; % = unadjusted prevalence rate; OR = odds ratio; CI = confidence interval; ref. = reference category. ^1^ Adjusted for age, residential region, urban/rural, immigrant status and type of health insurance. Note: The ORs are derived from separate regression models (analysis step 1); the *p*-values for the interaction term are derived from a common regression model including both age groups (analysis step 2).
